# Outcome Measures in Alveolar Ridge Augmentation in the Edentulous Maxilla: A Systematic Review and COSMIN Analysis

**DOI:** 10.1111/clr.70060

**Published:** 2026-02-24

**Authors:** Muhammad H. A. Saleh, Hamoun Sabri, Parham Hazrati, Hom‐Lay Wang

**Affiliations:** ^1^ Department of Periodontics and Oral Medicine University of Michigan School of Dentistry Ann Arbor Michigan USA; ^2^ Center for Clinical Research and Evidence Synthesis in Oral Tissue Regeneration (CRITERION) Ann Arbor Michigan USA

**Keywords:** bone regeneration, clinician‐reported outcomes, COSMIN, edentulism, edentulous maxilla, patient satisfaction, patient‐reported outcome measures

## Abstract

**Objectives:**

This systematic review evaluated the current outcome measures following alveolar ridge augmentation (ARA) in the edentulous maxilla. The secondary objective was to determine the validity of the identified patient‐reported outcome measures (PROMs) through standard analysis and methodology.

**Materials and Methods:**

A systematic review was conducted. Databases including MEDLINE, Embase, Scopus, and Cochrane Central were searched in April 2024, limited to studies published in the last 10 years. Inclusion criteria were prospective clinical studies with at least 10 patients per treatment arm, edentulous maxilla, and ARA procedures. The quality and validity of patient‐reported outcomes (PROs) were assessed using the COnsensus‐based Standards for the selection of health status Measurement INstruments (COSMIN) checklist, which systematically evaluates their reliability, validity, and responsiveness.

**Results:**

Of 1426 articles, 14 studies were included, with 11 unique study cohorts. Only 45.45% of the studies reported PROMs. The COSMIN analysis indicated that the Dutch version of the Oral Health Impact Profile (OHIP‐49NL) and the Denture Satisfaction Questionnaire showed strong psychometric properties. However, the Patient Satisfaction and the Change in Psychology Questionnaire and Patient's Overall Satisfaction with Denture Questionnaire exhibited significant shortcomings.

**Conclusion:**

Future research should focus on improving the validity of PROMs incorporating ARA‐specific outcome measures and adopting a dual‐phase approach for assessing early healing and long‐term PROMs to enhance study consistency and reliability.

## 1 Introduction

Edentulism poses significant challenges both functionally and psychologically (Emami et al. 2013; Polzer et al. 2010). This condition affects mastication, speech, and aesthetics, leading to a profound impact on the quality of life and overall health (Emami et al. 2013; Polzer et al. 2010). Complete edentulism is particularly prevalent among the elderly, with studies showing that a considerable proportion of the aging population suffers from this condition. A recent population‐based study indicated that around 13.54% of the over‐65‐year‐old US population suffers from complete edentulism (Vemulapalli et al. 2024). Tooth loss often leads to bone resorption, further complicating dental rehabilitation efforts (Gupta et al. 2019).

To address the functional and aesthetic problems caused by complete edentulism, dental implants have become a preferred solution. Dental implants offer a more stable and durable alternative to conventional dentures, significantly improving patients' oral health‐related quality of life (OHRQoL) (Gonçalves et al. 2022). However, in atrophic ridges, the condition can be more complex due to insufficient bone volume and density, often necessitating additional procedures such as alveolar ridge augmentation (ARA) to ensure the stability and success of the implants. Techniques like guided bone regeneration (GBR) and maxillary sinus augmentation are commonly employed to enhance bone volume in the maxillary region (Urban et al. 2017, 2021; Aghaloo et al. 2016).

Outcome assessments encompass survival, clinical outcome assessments (COAs), and biomarkers. A COA refers to any evaluation susceptible to influence by human decision‐making, judgment, or motivation. There are four categories of COAs: clinician‐reported outcomes (ClinROs), where a trained clinician rates the patient's status using professional judgment (not a direct biomarker); observer‐reported outcomes (ObsROs), where a non‐clinician observer (e.g., caregiver) records what they notice without needing specialized training; performance outcomes (PerfOs), where the patient completes a standardized task and trained staff quantify the result using preset rules with minimal rater judgment; and patient‐reported outcomes (PROs), where patients themselves directly report their symptoms, functioning, or health status without interpretation by others (Walton et al. 2015).

ClinROs are healthcare professional‐generated outcomes that observe a patient's health condition and provide an assessment based on clinical judgment or interpretation of observable signs, physical manifestations, and behaviors related to a disease or condition (Powers III et al. 2017). ClinROs include three types: (1) ClinRO readings: binary yes/no judgments of observed features (e.g., presence or absence of suppuration, bleeding on probing); (2) ClinRO ratings: multi‐level scores using standardized scales (e.g., severity of inflammation rated 0–3); and (3) clinician global assessments (CGAs): overall clinical judgments of a patient's health (e.g., “Is the site healed? Is this patient stable?”). When patients can directly assess the measured concept, PROs are preferable to ClinROs. Regardless of who assesses it, the measurement should be significant to the patient's experience (Saleh et al. 2026).

The importance of PROs lies in their ability to capture the patient's perspective on their treatment outcomes, providing valuable insights into the subjective benefits of medical interventions. Unfortunately, traditional clinical research has often overlooked aspects of care that matter most to patients. Evidence shows that there is very limited focus on PROs (Mounssif et al. 2023; Douglas‐de‐Oliveira and Chen 2023). This has resulted in a gap in understanding the full impact of treatments from the patient's viewpoint. However, recent attempts have been made to incorporate PROs (Douglas‐de‐Oliveira and Chen 2023; Avila‐Ortiz et al. 2023; Kofina et al. 2023; Messias et al. 2023) although this practice is still emerging. Integrating PROs is increasingly important as patient satisfaction is becoming a key determinant of success in dental treatments (Douglas‐de‐Oliveira and Chen 2023).

Patient‐reported outcome measures and clinician‐reported outcome measures are the tools used to evaluate PROs and ClinROs, respectively. For a given PRO or ClinRO, multiple “tools” may be found. For example, peri‐implant bone changes can be assessed using various tools, such as intra‐oral radiographs, panoramic radiographs, and cone beam computed tomography. Likewise, the PRO of satisfaction with treatment may be evaluated using different validated questionnaires, each serving as a PROM (Powers III et al. 2017).

Considering the mentioned concepts, this systematic review aims to comprehensively evaluate the current outcome measures implemented to assess ARA procedures in patients with an edentulous maxilla. Recognizing the importance of understanding variations in clinical use and potential gaps in evaluation, we aim to evaluate the outcomes and the tools employed for measuring outcomes in clinical research and trial settings, with a particular emphasis on assessing the validity of the identified PROMs through a standardized methodology.

## 2 Materials and Methods

### 2.1 Study Design, Protocol, and Registration

This study was conceptualized as a systematic review and *COnsensus‐based Standards for selecting health status Measurement INstruments* (COSMIN) analysis (Prinsen et al. 2018). A priori protocol was registered through the International Prospective Register of Systematic Reviews (PROSPERO) portal (CRD42024520298). The study was conducted in accordance with the Preferred Reporting Items for Systematic Reviews and Meta‐Analyses (PRISMA) statement (Page et al. 2021) (Table S1). Institutional Review Board (IRB) approval was deemed not required as this study exclusively utilized previously published data.

### 2.2 PICOST Elements and Focused Question

The following PICOST elements were defined to formulate the focused question.

*Population*: Adult patients (human) with a fully edentulous maxilla.

*Intervention*: ARA with various surgical techniques and grafting materials for at least one dental implant rehabilitation site.

*Comparison*: Not applicable.

*Outcomes*: Outcome measures like PROMs to assess PROs such as pain, discomfort, quality of life, overall satisfaction; other COAs like ClinROs, ObsROs, PerfOs; and other outcome measures such as implant survival and biomarkers.

*Study design*: Any prospective clinical study with at least 10 patients per treatment arm (such as prospective randomized or non‐randomized controlled clinical trials and prospective case series).

*Timeframe*: articles published between 2013 and 2024 were considered.

Based on the framework mentioned, the following focused question was considered:

In prospective clinical studies with at least 10 patients per treatment arm, published in the last 10 years, on patients with an edentulous maxilla requiring ARA prior to implant placement, what are the common outcome measures used to assess and report the treatment outcomes?

### 2.3 Eligibility Criteria

#### 2.3.1 Inclusion Criteria

- Prospective clinical studies with at least 10 patients.
- Edentulous maxilla.
- ARA procedures with various grafting materials and techniques with or without sinus floor elevation prior to implant placement.
- Studies reporting outcome measures.
- Articles published in any language (no language limitations).

#### 2.3.2 Exclusion Criteria

- Retrospective studies, case reports, any type of reviews, book chapters, commentaries, and technical notes, as well as pre‐clinical studies (animal, in vitro studies, etc.)
- Studies with a patient cohort of less than 10 subjects.

### 2.4 Search Strategy

A systematic search strategy was developed and conducted in MEDLINE (through PubMed), Embase, Scopus, and the Cochrane Central Register of Controlled Trials on April 5, 2024, with a limitation to studies published in the last 10 years.

No restrictions were assigned regarding the journal or the language used. However, a 10‐year time frame limitation from 2013 to the date of the search, April 5th, 2024, was applied. Next, the search results (after duplicate removal and cross‐reference checks in additional reports and citation lists) were complemented with a manual hand search and screening of the following journals: *Journal of Dental Research, Journal of Oral Rehabilitation, Journal of Clinical Periodontology, Journal of Periodontology, Clinical Oral Investigations, Journal of Stomatology, Oral and Maxillofacial Surgery, International Journal of Periodontics* and *Restorative Dentistry, Journal of Oral and Maxillofacial Surgery, International Journal of Oral and Maxillofacial Surgery, British Journal of Oral and Maxillofacial Surgery, Journal of Prosthodontics, Journal of Prosthetic Dentistry, International Journal of Prosthodontics, Journal of Advanced Prosthodontics, Clinical Oral Implants Research, Journal of Oral Implantology, International Journal of Oral Implantology, International Journal of Oral and Maxillofacial Implants, and Clinical Implant Dentistry and Related Research*. The complete search strategy corresponding to each of the databases queried is presented in Table 1.

**TABLE 1:** Comprehensive search strategy used in this systematic review.

		Last access date
*Databases*		
PubMed	((“maxilla*”[All Fields] OR “upper jaw”[All Fields]) AND (“edent*”[All Fields] OR “all‐on‐four”[All Fields] OR “all‐on‐x”[All Fields] OR “overdenture”[All Fields]) AND (“bone augmentation”[All Fields] OR “bone regeneration”[All Fields] OR “guided bone regeneration”[All Fields] OR “ridge augmentation”[All Fields])) AND (2013:2024[pdat])	04‐05‐2024
Embase	#1: (‘maxilla*’ OR ‘upper jaw’) #2: (‘edent*’ OR ‘all‐on‐four’ OR ‘all‐on‐x’ OR ‘overdenture’ OR ‘overlay denture’) #3: (‘bone augmentation’ OR ‘bone regeneration’ OR ‘guided bone regeneration’ OR ‘ridge augmentation’) #4: [2013–2024]/py #1 AND #2 AND #3 AND #4	04‐05‐2024
Scopus	TITLE‐ABS‐KEY ((maxilla* OR “upper jaw” OR “maxillary bone” OR “maxillary arch”) AND (“full arch” OR “fully edentulous” OR “complete edentulism” OR “all‐on‐four” OR “all‐on‐x” OR (“denture, overlay” OR (“denture” AND “overlay”) OR “overlay denture” OR “overdenture” OR “overdentures”) OR edent*) AND (“bone augmentation” OR “bone regeneration” OR “guided bone regeneration” OR “alveolar ridge augmentation” OR “ridge augmentation” OR gbr)) AND PUBYEAR AFT 2012	04‐05‐2024
*Register*		
Cochrane Central Register of Controlled Trials (CENTRAL)	(maxilla* OR “upper jaw”) in Title Abstract Keyword AND (edent* OR “all‐on‐four” OR “all‐on‐x” OR “overlay denture” OR “overdenture”) in Title Abstract Keyword AND (“bone augmentation” OR “bone regeneration” OR “guided bone regeneration” OR “ridge augmentation” OR GBR) in Title Abstract Keyword with Cochrane Library publication date Between Jan 2013 and Apr 2024	04‐05‐2024

### 2.5 Study Selection Process

The output of all the databases searched was entered into the Endnote software (version X9, Clarivate Analytics, Philadelphia, PA, USA). After duplicate removal, the titles and abstracts of all articles were screened by two authors (H.S., P.H.). The full texts of potentially relevant studies were retrieved and reviewed based on eligibility criteria by the same independent reviewers. To ensure calibration and consistency between the reviewers, an inter‐examiner reliability test (using Cohen's Kappa test) was performed on the first 100 screened articles to ensure that the reviewers achieved a minimum agreement of at least 85% before proceeding with the main title/abstract screening process. Any disagreement at this stage was resolved with consultation with a senior author (M.H.A.S.). Subsequently, Kappa values were calculated at each screening and eligibility step, and these values are reported in the PRISMA diagram to provide transparency.

### 2.6 Data Collection, Management, and Analysis

Studies that met the inclusion criteria underwent data collection by two independent reviewers (H.S., P.H.). A standardized data collection form, based on PRISMA guidelines, was developed to ensure consistency and rigor in data extraction. The form captured:

*Bibliographic information*: Author names, publication year, journal, and study setting.

*Study design*: Type of study (e.g., randomized controlled trial (RCT), prospective cohort).

*Ridge augmentation details*: Type of augmentation performed, materials used, and whether the procedure was accompanied by other interventions (e.g., sinus floor elevation).

#### 2.6.1 Outcome Measures

The form was pilot tested on a subset of five studies to ensure clarity and consistency before proceeding with full data extraction. Any missing data that potentially contributed to this systematic review was requested directly from the authors via email. Discrepancies during data extraction were resolved through discussion between the reviewers or consultation with a senior author (M.H.A.S.).

All descriptive and statistical analyses were performed by one author (H.S.) using Python software (version 3.11, Python Software Foundation, Wilmington, Delaware, USA). Specifically, the ‘Matplotlib’ (Tosi 2009) and ‘pandas’ (McKinney 2011) packages were used. More specifically, the ‘Matplotlib’ library was used for creating plots, and the ‘pandas’ library was employed for organizing, cleaning, and summarizing tabular data. To assess inter‐examiner reliability during the screening and eligibility steps, Cohen's Kappa values were calculated.

### 2.7 Quality Assessment and COSMIN Analysis

Measurement properties and the risk of bias in the development of included PROMs were assessed using the COSMIN Taxonomy. The following measurement properties were evaluated, with their definitions, as defined by Mokkink et al. (2018) and methods of assessment detailed below:

- *Content validity*: Assessed the extent to which a PROM reflects the construct it intends to measure, focusing on relevance, comprehensiveness, and comprehensibility. Evaluations included interviews with target populations and expert panels, considering item relevance, construct coverage, and clarity of instructions.
- *Structural validity*: Determined the alignment of PROM scores with their hypothesized structure, using methods such as factor analysis, item response theory (IRT), or Rasch modeling.
- *Internal consistency*: Examined the interrelation among items within a scale, assessed via Cronbach's alpha after verifying unidimensionality through structural validity.
- *Reliability*: Assessed the consistency of PROM scores under stable conditions, using test–retest reliability or inter‐rater reliability, depending on the study context.
- *Criterion validity*: Evaluated correlations between PROM scores and gold‐standard measures to confirm accuracy in reflecting the intended construct.
- *Hypotheses testing for construct validity*: Assessed predefined expectations of relationships between PROM scores and other variables. Significant correlations supported construct validity.
- *Responsiveness*: Evaluated the ability of a PROM to detect clinically meaningful changes using metrics such as effect sizes or standardized response means.

Two authors (H.S., P.H.) independently assessed these properties. Discrepancies were resolved through consensus meetings, achieving an inter‐examiner agreement of 83% (Cohen's Kappa).

**FIGURE 1:**
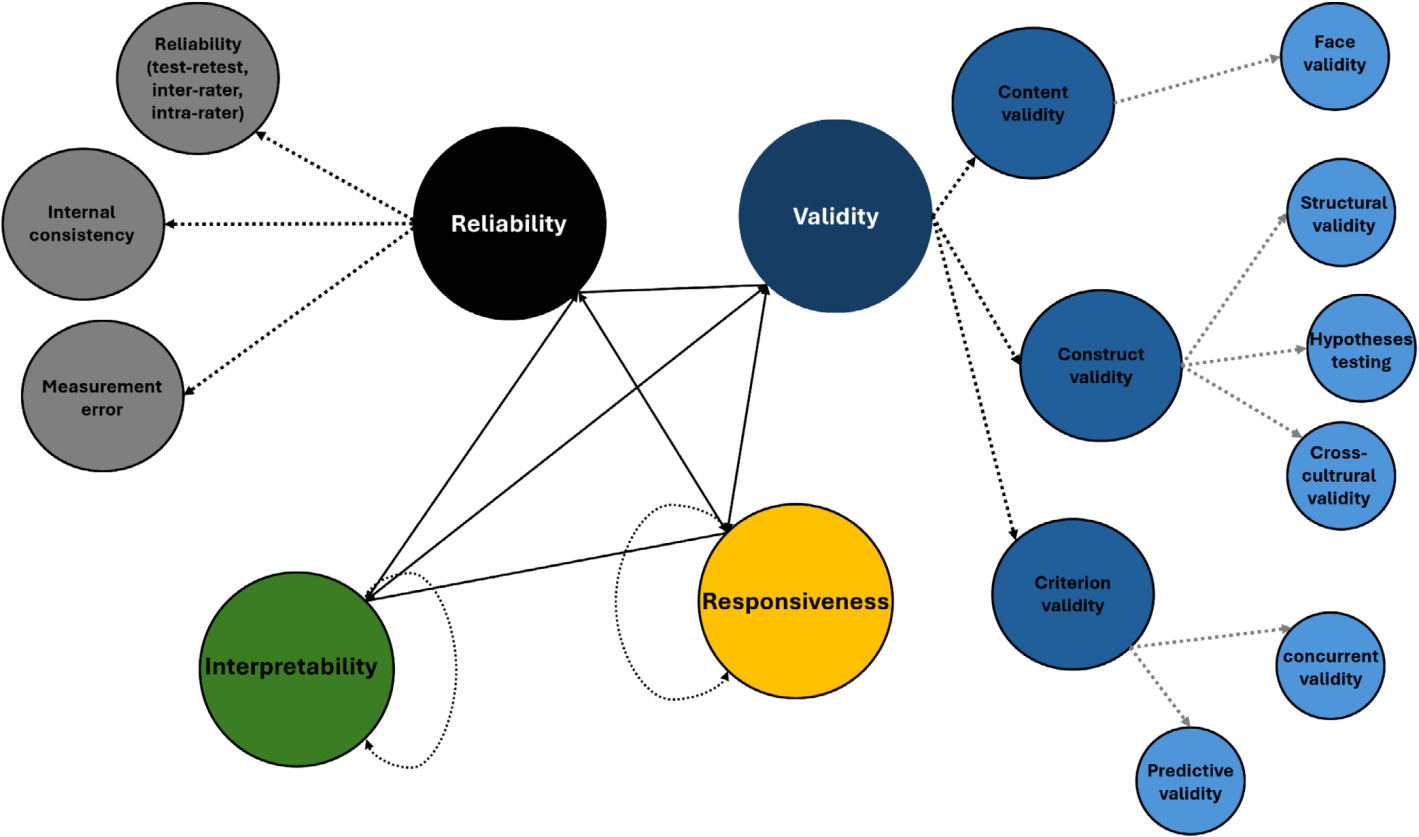
Network plot of the COSMIN analysis domains. The tool consists of four major domains (larger nodes): reliability, validity, responsiveness, and interpretability, which are interlinked with solid network lines. Each of these domains includes sub‐categories (depicted as smaller circles).

The quality of evidence for each property was summarized as sufficient, insufficient, or indeterminate using COSMIN criteria (Mokkink et al. 2018). Summary ratings for each property were determined using the lowest rating across property criteria (i.e., “worst score counts”), as specified by COSMIN guidelines; properties that were not reported were deemed inadequate, and the quality of the evidence was listed as “not applicable.” The data for each PROM was summarized independently. Figure 1 depicts the network plot of the performed COSMIN analysis and the implemented domains to assess the quality of PROMs. Also, Figure S1 summarizes the flowchart of the COSMIN methodology followed in this study.

## 3 Results

### 3.1 Study Selection

**FIGURE 2:**
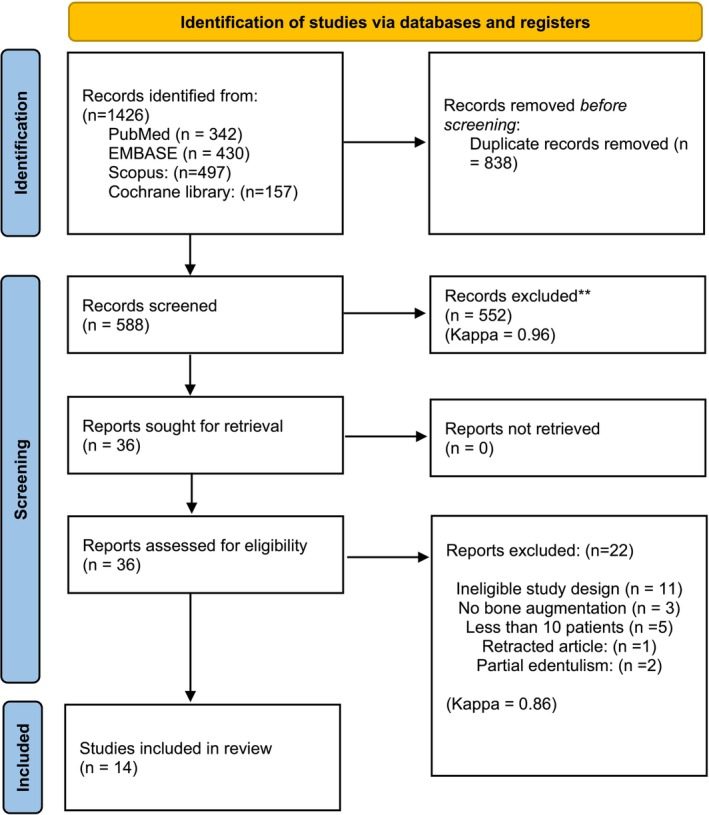
The PRISMA flowchart of study identification, screening, and inclusion of the systematic search strategy (agreement between the two examiners is reported as Kappa values).

The initial search strategy revealed 1426 articles in total. Out of 37 records that were selected for full‐text assessment, a total of 14 studies were finally included (Urban et al. 2017; Slot et al. 2016, 2023; Caramês et al. 2022; Wortmann et al. 2021, 2019; Putters et al. 2019, 2018; Grandi et al. 2019; Wu et al. 2015; Castagna et al. 2013; Marković et al. 2022; Aludden et al. 2024; Hernández‐Alfaro et al. 2013). Out of fourteen, 3 studies (Slot et al. 2023; Wortmann et al. 2021, 2019) were follow‐up studies of a previously published cohort. Therefore, the number of unique study cohorts included was 11. Inter‐examiner agreement kappa score for title/abstract review and for full‐text review was 0.96 (95% CI: 0.88–1.0) and 0.86 (95% CI: 0.79–0.95), respectively. The reason for the exclusion of the articles that did not meet the eligibility criteria is presented in Table S2. Also, the PRISMA diagram of the study selection process is depicted in Figure 2.

### 3.2 Characteristics of Included Articles

The articles included have been published between 2013 and 2024. Ten trials (Slot et al. 2016, 2023; Wortmann et al. 2021, 2019; Putters et al. 2019, 2018; Wu et al. 2015; Castagna et al. 2013; Marković et al. 2022; Aludden et al. 2024) were conducted in academic (university) settings, whereas four (Urban et al. 2017; Caramês et al. 2022; Grandi et al. 2019; Hernández‐Alfaro et al. 2013) studies were carried out in private practices. Among the included studies, eight were RCTs (Slot et al. 2016, 2023; Wortmann et al. 2021, 2019; Grandi et al. 2019; Marković et al. 2022; Aludden et al. 2024; Putters et al. 2018). One was a prospective controlled trial (Castagna et al. 2013), and five were prospective case‐series (Urban et al. 2017; Caramês et al. 2022; Putters et al. 2019; Wu et al. 2015; Hernández‐Alfaro et al. 2013). In ten studies (Urban et al. 2017; Slot et al. 2016, 2023; Wortmann et al. 2021, 2019; Putters et al. 2019; Grandi et al. 2019; Castagna et al. 2013; Aludden et al. 2024; Hernández‐Alfaro et al. 2013), simultaneous sinus floor elevation was also performed using the ridge augmentation procedure. The number of implants placed varied across the studies. Four studies (Slot et al. 2016, 2023; Caramês et al. 2022; Grandi et al. 2019) employed a four‐implant protocol, while six implants were placed in three others (Wu et al. 2015; Marković et al. 2022; Aludden et al. 2024). The remaining studies utilized a range of implant numbers (4 to 8) (Wortmann et al. 2021, 2019; Putters et al. 2019, 2018; Castagna et al. 2013; Hernández‐Alfaro et al. 2013; Urban et al. 2017). Regarding the augmentation protocols, five trials used various grafting materials mixed with intra‐oral autogenous bone (Urban et al. 2017; Slot et al. 2016, 2023; Aludden et al. 2024; Hernández‐Alfaro et al. 2013). One study used xenografts in conjunction with platelet‐rich fibrin (PRF) (Caramês et al. 2022), five studies opted for extraoral autogenous bone grafts (calvarial, iliac crest, or fibula grafts) (Wortmann et al. 2021, 2019; Wu et al. 2015; Castagna et al. 2013; Putters et al. 2018), and two studies used xenogeneic bone graft material (Grandi et al. 2019; Marković et al. 2022). The summary of the bibliographic information, methodology, treatment arms, study conclusions, and implemented PROMs and other outcome measures in the included studies is summarized in Table 2.

**TABLE 2:** Main characteristics of the included studies.

Author, year, country, study design, funding	Study groups/implant planning	*N* patients/implants/site/implant plan	BA method and material used	SFE?	Biomarkers ClinROs	PROMs/PerfOs	Conclusions
Slot et al. (2023), The Netherlands, RCT, Astra Tech AB, Mölndal, Sweden (10‐year follow‐up study of Slot et al. (2016))	Bar supported overdenture on 4 implants in anterior maxilla Bar supported overdenture on 6 implants in anterior maxilla Implants: OsseoSpeedTM 4.0S dental implants, Astra Tech AB, Mölndal, Sweden	25/100 (anterior maxilla) 25/150 (anterior maxilla)	Autologous bone from the maxillary tuberosity + organic bovine bone (Bio‐Oss, Geistlich Pharma AG, Wolhusen, Switzerland) + Resorbable membrane (Bio‐Gide, Geistlich Pharma AG, Wolhusen, Switzerland)	No Yes, bilateral SFE in 9 patients	A. Peri‐implant bone‐level changes B. Implant and overdenture survival C. Complications D. Presence of plaque, calculus and bleeding E. Degree of peri‐implant inflammation F. Probing depth	A. “Chewing ability” questionnaire B. The patient's overall satisfaction with the denture, expressed on a 10‐point rating scale.	Similar and favorable outcomes are seen in bar‐supported maxillary overdentures on either four or six anteriorly placed implants after a 10‐year evaluation period.
Caramês et al. (2022), Portugal, Prospective Clinical Study, No	Immediate‐loading full‐arch rehabilitation/4 implants; in the former positions of the lateral incisors and first premolars for immediate metal‐reinforced fixed complete dentures. Implants: Straumann Bone Level Tapered Implants, Basel, Switzerland.	18/72 (anterior maxilla)	Particulate xenograft (Bio‐Oss small particles, Geistlich AG, Wolhusen, Switzerland) + PRF membrane	No	A. Radiographic linear horizontal bone gain: 3.24 mm (95% CI: 2.85–3.64) B. Augmented bone stability C. Complications: 0% D. Implant survival: 98.61%	NR	PRF associated with a xenograft seems to promote an effective horizontal bone gain. Randomized clinical trials are needed to confirm the benefits of this surgical approach.
Wortmann et al. (2021), Wortmann et al. (2019), and Putters et al. (2018), The Netherlands, RCT, NA	Anterior iliac crest bone graft Calvarial bone graft Implants were placed 4 months after grafting.	10/44/NR 10/44/NR	Monocortical iliac crest bone graft Calvarial bone grafts	Yes, Bilateral maxillary sinus elevation surgery with cancellous bone.	A. Implant survival: recorded as loose or lost implants. B. MicroCT of biopsies (tissue mineral density (TMD), bone mineral density (BMD) and bone volume fraction (BVF)) C. Histology and histomorphometry (bone percentage (Bp), osteoid percentage (Op), and osteocyte number per volume (OcN/Ba)) D. donor site morbidity (pre‐operative, early and late) E. Hospitalization period (days)	A. Patient satisfaction. B. VAS for pain levels. C. VAS for 12 months satisfaction (0 ‘bad outcome’ to 10 ‘a good outcome’) D. OHIP‐49NL	Both donor sites, that is, anterior iliac crest and calvarial bone, are well suited to provide a reliable and stable basis for implant placement 4 months after grafting with mineral density, porosity, and resorption rate in favor of calvarial bone grafts.
Putters et al. (2019), The Netherlands, Prospective Clinical Study, No	Immediate implant placement in calvarial graft. In 5 patients: 4 implants & in 8 patients: 6 implants Implants: diameter 4.0 mm, length 11.5 mm; Biomet Nanotite Certain Tapered Implant, Biomet 3i, Dordrecht, The Netherlands	13/68/NA	Outer table calvarial bone blocks	Yes, bilateral window sinus floor elevation. Material: Calvarial bone mass.	A. Intraoral wound dehiscence B. Signs of infection (swelling, redness, fistulae) C. Signs of peri‐implant bone loss D. Signs of resorption around screw heads used for fixing bone blocks E. Signs of inflammation (granulomatous tissue, bone graft loss) F. Peri‐implant mucositis (Patient level) G. Peri‐implantitis (Patient level) H. Loss of implants I. Gingival hyperplasia under the bar construction. J. Need for additional surgical pro‐ cedures (correction hyperplasias, bone recontouring, removal of implants) K. Peri‐implant bone loss in OPG	A. VAS for pain level.	Immediate dental implant placement in calvarial bone grafts to rehabilitate severely resorbed maxilla is technically feasible and seems to have a high success rate.
Grandi et al. (2019), Italy and Lebanon, RCT, No	Tilted trans‐sinus implants inserted without simultaneous bone grafting Tilted trans‐sinus implants inserted with simultaneous bone grafting and sinus elevation	16/76 (18 trans‐sinus)/NA 16/72 (23 trans‐sinus)/NA	Immediately loaded fixed restoration supported by 4 or 6 implants receiving at least one trans‐sinus implant without simultaneous bone grafting Immediately loaded fixed restoration supported by 4 or 6 implants receiving at least one trans‐sinus implant with simultaneous bone grafting and sinus elevation Implants: trans‐sinus implants: diameter: 4 mm, lengths: 20, 22, 24 and 26 mm model: JDNasal, JDentalCare, Modena, Italy other implants: JDNasal, JDentalCare, Modena, Italy	No. Sinus membrane was ruptured. Yes. Lateral window technique. Material: xenograft (Bio‐Oss, Geistlich Pharma, Wolhusen, Switzerland)	A. Prosthesis failure B. Implant failure C. Peri‐implant bone‐level changes D. Complications	Patient reported complaints (sinusitis)	No statistically significant differences were observed between subjects treated with tilted trans‐sinus implants without simultaneous bone‐grafting or with sinus elevation procedures supporting cross‐arch immediately loaded fixed prostheses in atrophic maxillae.
Urban et al. (2017), Hungary and USA, Case series, University of Michigan graduate student research funds.	Vertical and/or Horizontal Guided Bone Regeneration in Combination with Maxillary Sinus Augmentation	16/122/NA 114 anodized TiUnite and 8 acid etched Steri‐Oss implants, Nobel Biocare, Gothenburg, Sweden: 1 case: 5 1 case: 6 3 cases: 7 9 cases: 8 2 cases: 9	1. Autogenous particulated bone or a 1:1 ratio of autogenous bone and anorganic bovine bone‐derived mineral (ABBM, Bio‐OssVR, Geistlich Pharma AG, Wolhusen, Switzerland) + 4 cases: Dense titanium reinforced non‐resorbable membrane (d‐PTFE; CytoplastTM Ti‐250 Titanium‐ Reinforced Membrane, Osteogenics Biomedical Inc., Lubbock, Texas) 4 cases: Titanium reinforced expanded polytetrafluoroethylene non‐resorbable membrane (e‐PTFE; GORE‐TEXVR Regenerative Membrane, Titanium‐Reinforced; W.L. Gore & Associates, Flagstaff, AZ) 3 cases: Resorbable membrane (GORE RESOLUTVR ADAPTVR LT Regenerative Mem‐ brane, W.L. Gore & Associates Inc., Flagstaff, AZ) 9 cases: Resorbable membrane (Bio‐GideVR Resorbable Bilayer Membrane, Geistlich Pharma AG, Wolhusen, Switzerland)	Yes. Lateral window SFE with autogenous particulated bone or a 1:1 ratio of autogenous bone and anorganic bovine bone‐derived mineral (ABBM, Bio‐OssVR, Geistlich Pharma AG, Wolhusen, Switzerland) and a resorbable collagen membrane.	A. Implant survival B. Bone gain C. Intraoperative/postoperative complications D. Peri‐implant bone loss	NR	Complete reconstruction of an atrophied maxilla can be successfully achieved by means of guided bone regeneration for horizontal and/or vertical bone gain including bilateral maxillary sinus augmentation using a mixture of anorganic bovine bone and autologous bone.
Wu et al. (2015), China, Prospective Clinical Study, Yes	Edentulous adult ectodermal dysplasia patients needing complete maxillary rehabilitation. 2 zygomatic implants +4 conventional implants for Implant‐supported fixed dentures	10/60/NR	Autologous iliac crest: 6 or fibula grafts: 4 in anterior maxilla + GBR GBR material: Bio‐Oss, Geistlich, Wolhusen, Switzerland + Bio‐Gide, Geistlich, Wolhusen, Switzerland	No	A. Probing depth (PD) B. sulcular bleeding index (SBI) C. Plaque index (PI) D. Gingival index E. Peri‐implant bone loss F. Prosthodontic complications and repairs G. Implant survival and success rate	A. Patient satisfaction questionnaire in 3 Parts (0 = unsatisfied; 1 = partially satisfied; 2 = fully satisfied.) Domains included: A. Patient satisfaction with: a. Facial contour b. Prosthesis esthetics c. Prosthesis function d. Pronunciation B. Change of psychology: a. Depression b. Self‐image c. Self‐esteem d. Social self‐confidence e. Partner f. Vocational factors	Oral function can be restored in adults with edentulous maxilla using a comprehensive and systematic treatment protocol involving psychological and oral education, ARA, implant placement, and denture fabrication. Despite these positive outcomes, ARA remains challenging in the anterior region of the edentulous maxilla.
Castagna et al. (2013), Brazil, CCT, No	Patients with generalized, severe atrophy of the edentulous maxilla receiving immediate total provisional prosthesis on temporary immediate implants.	12/NA/48 (Provisional) 4/30/NA (no provisionalization) (1) 4 provisional implants (temporary, all‐acrylic bridge with four temporary abutments connected to the provisional implants was placed 2–7 days after surgery) (2) Receiving only graft without any immediate implants. Provisional implants: Intralock 2.0; Intra‐ Lock1 System, Boca Raton, FL, USA All patients received 7.5 (12: 8, 4: 6) final implants after an average of 6 months and received a permanent metal ceramic full bridge. Implants: 10–12 mm; diameter, 3.3–4.1 mm, Straumann, Walden, Switzerland	Iliac corticocancellous blocks	Bilaterally with lateral window technique. Material: Particulated corticocancellous chips, milled with the Quetin Bone Mill.	A. Vertical and horizontal bone gain. B. Periodontal mobility test of implants. C. Implant success. D. Complications.	NR	The results suggest that the use of total provisional prostheses on temporary immediate implants meets the aesthetic demands required but should be used with care and in special cases.
Slot et al. (2016), The Netherlands, RCT, Astra Tech AB, Molndal, Sweden.	All on four All on six	1–24/NA/96 2–22/NA/132 Both receiving an overdenture retained by bars. Implants: OsseoSpeedTM 4.0 S dental implants, Astra Tech AB, Mondal, Sweden Length: at least 11 mm Diameter: at least 4 mm	Performed in sites with dehiscence or fenestration. Material: Bone harvested from the maxillary tuberosity + Bovine‐derived xenograft (Geistlich Bio‐Oss, Geistlich, Wolhusen, Switzerland)	Yes, if the most distally placed implants (usually in the six implants group) were partially placed in the anterior part of the maxillary sinus.	A. Changes in the marginal bone level. B. Implant survival. C. Overdenture survival. D. Plaque index E. Presence of calculus F. Gingiva index G. Sulcus bleeding index H. Pocket probing depth I. Peri‐implant mucositis J. Peri‐implantitis.	A. Patients' satisfaction questionnaire. This questionnaire focused on complaints and consisted of 54 items and was divided into six scales: Nine items concerning functional problems of the upper denture. Eighteen items concerning functional complaints in general. Three items concerning facial aesthetics; Three items concerning accidental lip, cheek and tongue biting (“neutral space”); Twelve items concerning aesthetics of the denture. The extent of each specific com‐ plaint could be expressed on a four‐point rating scale (0 = no com‐ plaints, 1 = little, 2 = moderate, 3 = severe complaints). B. “Chewing ability” questionnaire In this questionnaire, patients gave their opinion about the ability to chew nine different kinds of food on a three‐point rating scale (0 = good, 1 = moderate, 2 = bad). The items were grouped into three scales, being soft food, tough food and hard food. C. Patients' overall satisfaction with the treatment expressed on a 10‐point VAS.	In patients with functional complaints of their maxillary denture, bar‐supported overdentures on four implants in the anterior maxillary region were not inferior to overdentures supported by six implants after 5 years of function. Implant survival and patient satisfaction were high, clinical parameters favorable, bone loss and complications to the denture were minor in both groups
Marković et al. (2022), Serbia, RCT, Straumann	All on 6, immediately loaded with provisional complete fixed denture All on 6, loaded 6 weeks after surgery with definitive prosthesis Implants: BLT SLActive Roxolid implants (Straumann)	1–12/72/NA 2–12/72/NA	In sites with dehiscence or fenestration. Material: Bovine‐derived xenograft (Geistlich Bio‐Oss, Geistlich, Wolhusen, Switzerland)	No	A. Insertion torque B. Implant stability using resonance frequency analysis (RFA) (ISQ) C. Complications. D. Clinician satisfaction (VAS)	A. OHIP‐19 B. Patients' satisfaction related to function, esthetics, and discomfort/pain by indicating a position along a 10 scale VAS.	Although both loading protocols could significantly improve oral health related quality of life in patients, immediate loading (according to the Straumann Pro Arch concept) could significantly reduce physical pain and psychological disability while providing higher patient satisfaction in terms of function and aesthetics of the prosthodontic restoration.
Aludden et al. (2024), Sweden, RCT, Geistlich Pharma AG, Wolhusen, Switzerland.	Horizontal GBR with a graft composition of: 1%–100% DBBM (100:0) on one side 2%–90% DBBM and 10% PAB (90:10) on the other side	1–18 sites 2–18 sites	Left or right side was randomized to receive a graft composed of DBBM:PAB in a ratio of 100:0 or 90:10. The grafts were placed on the buccal aspect of the alveolar process from the region of the central incisor to the region of the second premolar densely packed under a resorbable collagen membrane (Bio‐Gide, Geistlich Pharmaceutical) fixed by titanium pins (Master Pin).	Yes‐ in 15 out of 18 patients (bilateral) (no details)	A. CBCT Linear changes B. CBCT Volumetric changes C. Histological assessment	NR	There were no additional effects of adding PAB to DBBM regarding bone formation, width changes, or volumetric changes after 10 months of graft healing following horizontal augmentation by the GBR principles in edentulous atrophic maxilla.
Hernández‐Alfaro et al. (2013), Spain, Prospective clinical study, No	6–10 implants loaded with fixed ceramic‐metal restorations	14/108/anterior and posterior	Autograft (Ramus) + small‐particle Bio‐Oss was applied to the buccal wall + Bio‐Gide membranes	Yes, “bilateral Lateral window SFE in all cases (A resorbable col‐lagen membrane) (Bio‐Gide, Geistlich Pharma) was positioned against the elevated membrane, and the underlying space was filled with demineralized bovine bone particles (Bio‐Oss, Geistlich Pharma).”	A. ISQ B. Volume change (CBCT)	NR	Results of this study suggest that the use of mandibular bone blocks in combination with biomaterials is an effective, reliable procedure for the rehabilitation of the severely resorbed maxilla. Significant volume increases and adequate stability of the augmented areas at reentry were found with CBCT analysis. The grafted bone provided sufficient mechanical support to permit provisionalization and immediate loading. This technique enabled the restoration of function and esthetics with a fixed rehabilitation at 4 months.

Abbreviations: ABBM: anorganic bovine bone‐derived material; ARA: alveolar ridge augmentation; BA: bone augmentation; BLT: bone level tapered; BMD: bone mineral density; BVF: bone volume fraction; Bp: bone percentage; CCT: controlled clinical trial; CI: confidence interval; ClinROs: clinician‐reported outcomes; DBBM: demineralized bovine bone material; d‐PTFE: dense polytetrafluoroethylene; e‐PTFE: expanded polytetrafluoroethylene; GBR: guided bone regeneration; ISQ: implant stability quotient; MicroCT: micro–computed tomography; NA: not available; NR: not reported; OcN/Ba: osteocyte number per bone area; OHIP‐49NL: 49‐item Oral Health Impact Profile, Dutch (Netherlands) version; OHIP‐19: 19‐item Oral Health Impact Profile; Op: osteoid percentage; OPG: orthopantomogram; PAB: particulate autogenous bone; PD: probing depth; PerfOs: performance outcomes; PI: plaque index; PRF: platelet‐rich fibrin; PROMs: patient‐reported outcome measures; RCT: randomized controlled trial; SBI: sulcular bleeding index; SFE: sinus floor elevation; TMD: tissue mineral density; VAS: visual analogue scale.

### 3.3 Qualitative Assessment

#### 3.3.1 Outcome Measures Used in the Included Studies

**FIGURE 3:**
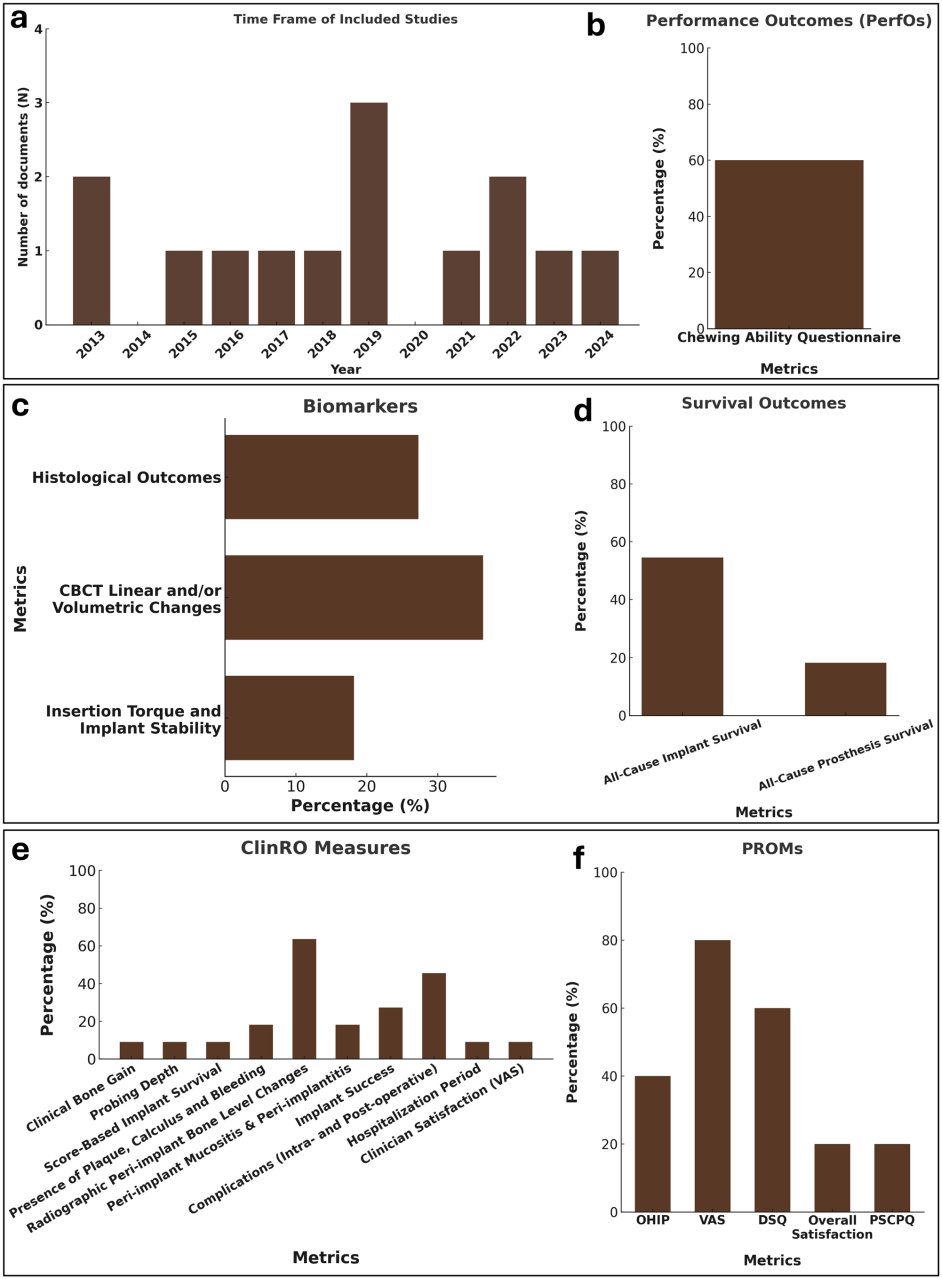
Multi‐panel figure of the characteristics of the included studies and identified measures. (a) Publication time frame of the included studies. (b) Performance outcomes (PerfOs). (c) Bar graph of biomarkers reported in the studies included. (d) Prevalence of survival outcomes. (e) Clinician‐reported outcome measures (ClinROs) and (f) patient‐reported outcome measures (PROMs). (ChA Q, chewing ability questionnaire; DSQ, Denture Satisfaction Questionnaire; OHIP, Oral Health Impact Profile; PSCPQ, Patient Satisfaction and Change of Psychology Questionnaire; VAS, Visual Analogue Scale).

Upon critical assessment of the selected studies in this systematic review, outcome measures were identified and categorized, as summarized in Table 2. Figure 3 summarizes the publication time frame of the included studies (a) as well as the frequency they were reported within the included trials (b–d).

#### 3.3.2 COAs Measures

##### 3.3.2.1 Patient‐Reported Outcome Measures (PROMs)

Only five (Slot et al. 2016; Wu et al. 2015; Marković et al. 2022; Wortmann et al. 2019; Putters et al. 2018) out of eleven unique trials assessed PROs. Six PROMs were identified as follows: (Table 3)

- *Oral Health Impact Profile (OHIP) [OHIP‐19E, OHIP‐49NL]*: The PRO assessed was quality of life. Two studies (Marković et al. 2022; Wortmann et al. 2019) (40%) used this tool in their protocol. While the original version of this questionnaire introduced by Slade and Spencer (1994) was developed for dentate individuals and consists of 49 questions, among the included studies in this review, one study used a shorter version of 20‐questions (Marković et al. 2022). These two questionnaires (OHIP‐49NL and OHIP‐19E) are customized for the edentulous maxilla. Moreover, the study by Wortmann et al. (2019), implemented a cross‐culturally validated Dutch version of the original 49‐item questionnaire.
- *Visual Analogue Scale (VAS)*: PROs assessed using VAS were pain and overall satisfaction and function. Among the articles included, 4 studies used this tool (Putters et al. 2019, 2018; Marković et al. 2022; Wortmann et al. 2019) (80%). Putters et al. (2018); Putters et al. (2019); and Marković et al. (2022) used a 10‐scale VAS to assess pain levels (from ‘no pain’ to ‘pain as bad as it could be’). These three studies as well as the study by Wortmann et al. (2019) also used a VAS scale corresponding to the overall satisfaction and function (from ‘very unsatisfied’ to ‘very satisfied’).
- *Denture Satisfaction Questionnaire*: This is a validated questionnaire (Vervoorn et al. 1988) which was used in three of the included trials (Slot et al. 2016, 2023; Wortmann et al. 2019) (60%). This consists of 8 domains (including functional complaints, vague denture complaints, aesthetic complaints, general satisfaction etc.).
- *Patient's overall satisfaction with denture*: The study by Slot et al. (2023) (20% of all included studies that reported PROMs) employed a modified questionnaire containing a 0 to 10 scale, indicating patients' overall satisfaction with the denture.
- *“Patient' satisfaction and change of psychology” questionnaire*: This questionnaire was used in one study (Wu et al. 2015) (20%). Unfortunately, the original source of this tool was not mentioned in the article. Generally, this tool compares before and after changes in patient satisfaction and changes in psychology. The satisfaction domain consists of facial contour, prosthesis esthetics, prosthesis function, and pronunciation. Moreover, the psychology domain includes depression, self‐image, self‐esteem, confidence, partner, and vocational factors. Each part is scored on a 3‐point scale of 0 (unsatisfied), 1 (partially satisfied), and 2 (fully satisfied).

**TABLE 3:** Description of included PROMs and identified PerfO (Chewing ability questionnaire) with the references in which the tool is used along with their original reference and full description.

PROM and studies used	Original reference	Description
Denture Satisfaction Questionnaire (Slot et al. 2023) (Slot et al. 2016)	Vervoorn et al. (1988)	1A. Functional complaints of the maxillary denture; this is the total score of 12 complaints concerning the upper denture 1B. functional complaints of the mandibular denture; this is the total score of 8 complaints concerning the lower denture 1C. vague denture complaints; this is the total score of 11 vague complaints not specifically related to the maxillary or mandibular denture 1D. aesthetic complaints: “too hollow”; this is the total score of 5 complaints related to a hollow face 1E. aesthetic complaints “Too bulbous”; this is the total score of 4 complaints related to a bulbous face 2 General satisfaction 3 Satisfaction with maxillary denture 4 Satisfaction with mandibular denture 5 Aesthetie satisfaction 6 Satisfaction with retention 7 Functional satisfaction 8 The denture satisfaction score
**“**Chewing ability” questionnaire (Slot et al. 2023) (Slot et al. 2016)	Stellingsma et al. (2005)	Patients' opinion about their ability to chew nine different kinds of food on a 3‐point rating scale (0 = good, 1 = moderate, 2 = bad). ‘Soft food’ (boiled vegetables and potatoes, crustless bread, minced meat) ‘Tough food’ (crusty bread, steak, Gouda cheese) ‘Hard food’ (apple, carrot, peanuts)
Patient's overall satisfaction with denture (Slot et al. 2023) (Slot et al. 2016)	Slot et al. (2023)	The patient's overall satisfaction with the denture, expressed on a 10‐point rating scale.
Oral Health Impact Profile‐19; OHIP‐19 (Marković et al. 2022)	Slade and Spencer (1994)	Included 20 questions and for each question the patient had to give a score. The frequency ranged between 1 (always) to 6 (never), and the questions were related to: Functional limitation Physical pain Physical disability psychological discomfort Social disability.
Visual Analogue Scale (VAS) for pain level/overall satisfaction/function (Putters et al. 2019) (Marković et al. 2022)	Miller and Ferris (1993) Johnson (2001)	The VAS consists of a 10 cm line, with two end points representing 0 (‘no pain’) and 10 (‘pain as bad as it could possibly be’).
“Patient Satisfaction and Change of Psychology” questionnaire (Wu et al. 2015)	The initial article was not clearly mentioned.	Before and after treatment changes compared: Questionnaire in 3 Parts (0 = unsatisfied; 1 = partially satisfied; 2 = fully satisfied.) A. Patient satisfaction a. Facial contour b. Prosthesis esthetics c. Prosthesis function d. Pronunciation B. Change of psychology a. Depression b. Self‐image c. Self‐esteem d. Social self‐confidence e. Partner f. Vocational factors
OHIP‐EDENT‐19 (Marković et al. 2022)	Allen and Locker (2002)	Contains 19 questions about oral function, chewing ability, and problems related to their dentures. The responses were coded from 1 to 5; a higher value indicates a more affirmative response.

##### 3.3.2.2 Clinician‐Reported Outcome Measures

- *Clinical bone gain*: Only one trial (Urban et al. 2017) (9%) measured the direct clinical bone dimensions using a caliper, as the ClinRO, at the baseline and the re‐entry surgery for implant placement.
- *Radiographic peri‐implant bone‐level changes*: This ClinRO was assessed as the mean of mesial and distal bone level changes observed in intra‐oral peri‐apical radiographs across seven studies (63.63%), including Slot et al., (in their 3‐year (Slot et al. 2016) and 10‐year (Slot et al. 2023) follow‐up studies), Grandi et al. (2019) (1‐year post‐loading), Urban et al. (2017) (1 to 10‐year post‐loading; annually) and Wu et al. (2015) (3‐year post surgery). Putters et al. (2019) used panoramic images instead of peri‐apical, but with the same criteria to measure the bone level changes.
- *Score‐based Implant survival*: In one study (9%) (Urban et al. 2017). Consensus of Pisa statement criteria for implant survival was used (Misch et al. 2008).
- *Presence of plaque, calculus and bleeding*: Only two trials (18.18%) reported these outcomes among the main outcomes (primary or secondary) of their study (Slot et al. 2023; Wu et al. 2015). Plaque index, Gingival index, sulcular bleeding index were defined using Mombelli et al. (1987) criteria. Modified Loe and Silness (1963) criteria was used for the degree of peri‐implant inflammation.
- *Probing depth*: Only one study (9%) assessed peri‐implant probing pocket depths as a main outcome (Wu et al. 2015). It should be noted that, in other studies where prevalence of peri‐implant diseases was assessed, this ClinRO was among one of the criteria and was indirectly included in the study.
- *Peri‐implant mucositis and peri‐implantitis*: Two trials (18.18%) reported diagnosis of peri‐implant diseases (Slot et al. 2023; Putters et al. 2019), where VIII European Workshop on Periodontology definitions (Lang and Berglundh 2011) were used.
- *Implant success*: Different criteria were considered for implant success in each of the three studies (27.27%) evaluating this ClinRO. Grandi et al. (2019) used the following criteria: successful implant function, stability, absence of infection, absence of radiolucency, absence of esthetic issues, satisfactory hygiene and patient‐confidence outcome. Moreover, Urban et al. (2017), used the criteria for success reported in the Consensus of Pisa statement 2008 (Misch et al. 2008). Furthermore, one study (Castagna et al. 2013) followed Albrektsson and Zarb's criteria (Albrektsson et al. 1986) for implant success (no mobility, no radiolucency, no more than 0.2 mm of bone loss annually after first year of placement, no signs and symptoms) (Albrektsson et al. 1986).
- *Complications (intra‐operative and post‐operative)*: Five studies (45.45%) reported complications (Caramês et al. 2022; Putters et al. 2019, 2018; Grandi et al. 2019; Wu et al. 2015). Caramês et al. (2022) defined this as: sensory disorder, infection, graft exposure, and dehiscence. Putters et al. (2018) assessed this outcome as: “donor site morbidity” after the surgery. This included reporting of any complications such as necrosis, flap dehiscence etc. pertinent to the donor site.
- *Hospitalization period (days)*: This ClinRO was only reported in one study (Putters et al. 2018) (9%) where calvarial bone grafts were harvested from one of the trial groups. the authors reported the number of days that each subject was hospitalized after graft harvesting.
- *Clinician satisfaction (via VAS)*: In one study (Marković et al. 2022) (9%), in addition to the overall patient satisfaction (via VAS), clinician's satisfaction was also assessed.

##### 3.3.2.3 Performance Outcome (PerfO) Measures

- *Chewing ability questionnaire*: Three studies included this questionnaire (60%) in their trials (Slot et al. 2016, 2023; Wortmann et al. 2019). This questionnaire queries patients about their ability to chew nine different kinds of food on a 3‐point rating scale (0 = good, 1 = moderate, 2 = bad). This consists of: (1) ‘soft food’ (boiled vegetables and potatoes, crustless bread, minced meat) (2) ‘tough food’ (crusty bread, steak, Gouda cheese) (3) ‘hard food’ (apple, carrot, peanuts).

##### 3.3.2.4 Observer‐Reported Outcome (ObsRO) Measures

None of the included studies used any ObsRO as an outcome measure.

#### 3.3.3 Survival Outcomes

- *All‐cause implant survival*: This outcome was defined as loose or lost implants and was assessed in six studies (Slot et al. 2023; Caramês et al. 2022; Wortmann et al. 2021; Grandi et al. 2019; Putters et al. 2018; Misch et al. 2008) (54.54%).
- *All‐cause prosthesis survival*: Two studies (18.18%) reported this outcome (Slot et al. 2023; Grandi et al. 2019). Grandi et al., considered a prosthesis failed if it was replaced by another prosthesis (Grandi et al. 2019).

#### 3.3.4 Biomarkers

- *Histological outcomes* Biopsies from the grafted sites were obtained in three trials (Wortmann et al. 2021; Putters et al. 2019; Aludden et al. 2024) (27.27%). In the study by Wortmann et al. (2021), and Putters et al. (2019), bone biopsies from the reconstructed maxillae were harvested at the 4‐month follow‐up as well as the donor sites at the baseline. Wortmann et al., obtained a high‐resolution micro‐CT and analyzed them for: the tissue mineral density (TMD, mg HA/cm^3^) The bone mineral density (BMD, mg HA/cm^3^) the bone volume fraction (BVF) (Wortmann et al. 2021). Also, both studies (Wortmann et al. 2021; Putters et al. 2019) performed histomorphometric analysis of bone percentage (Bp), osteoid percentage (Op), and osteocyte number per volume (OcN/Ba). Aludden et al. (2024) also collected bone biopsies and assessed the following: Total area of the grafted area, Total bone area, Total area of bovine bone graft material and the Total area of non‐mineralized tissue (NMT).
- *CBCT linear and/or volumetric changes*: Four trials (36.36%) assessed CBCT outcomes. Caramês et al. (2022) obtained pre‐, post‐ and 12‐month CBCT scans. Similarly, Castagna et al. (2013), obtained CBCT scans at baseline and before the re‐entry (implant placement at 6 months), where they measured and compared horizontal and vertical bone dimensions. Aludden et al. (2024) obtained 3 CBCTs pre‐, post‐operatively as well as at the 10‐month follow‐up to assess the linear and volumetric changes. Same approach was used in another study (Hernández‐Alfaro et al. 2013) but with the last scan obtained between 14 and 16 weeks post‐operatively.
- *Insertion torque and implant stability*: This was assessed only by two studies (18.18%). A ratchet device was used in one study (Marković et al. 2022) to assess implant insertion torque. In two of the studies (Marković et al. 2022; Hernández‐Alfaro et al. 2013) resonance frequency was recorded using the Osstell Mentor (Osstell, Gothenburg, Sweden) (Marković et al. 2022; Hernández‐Alfaro et al. 2013), and Penguin (PenguinRFA, Osstell) (Marković et al. 2022).

### 3.4 COSMIN Analysis on Included PROMs

**FIGURE 4:**
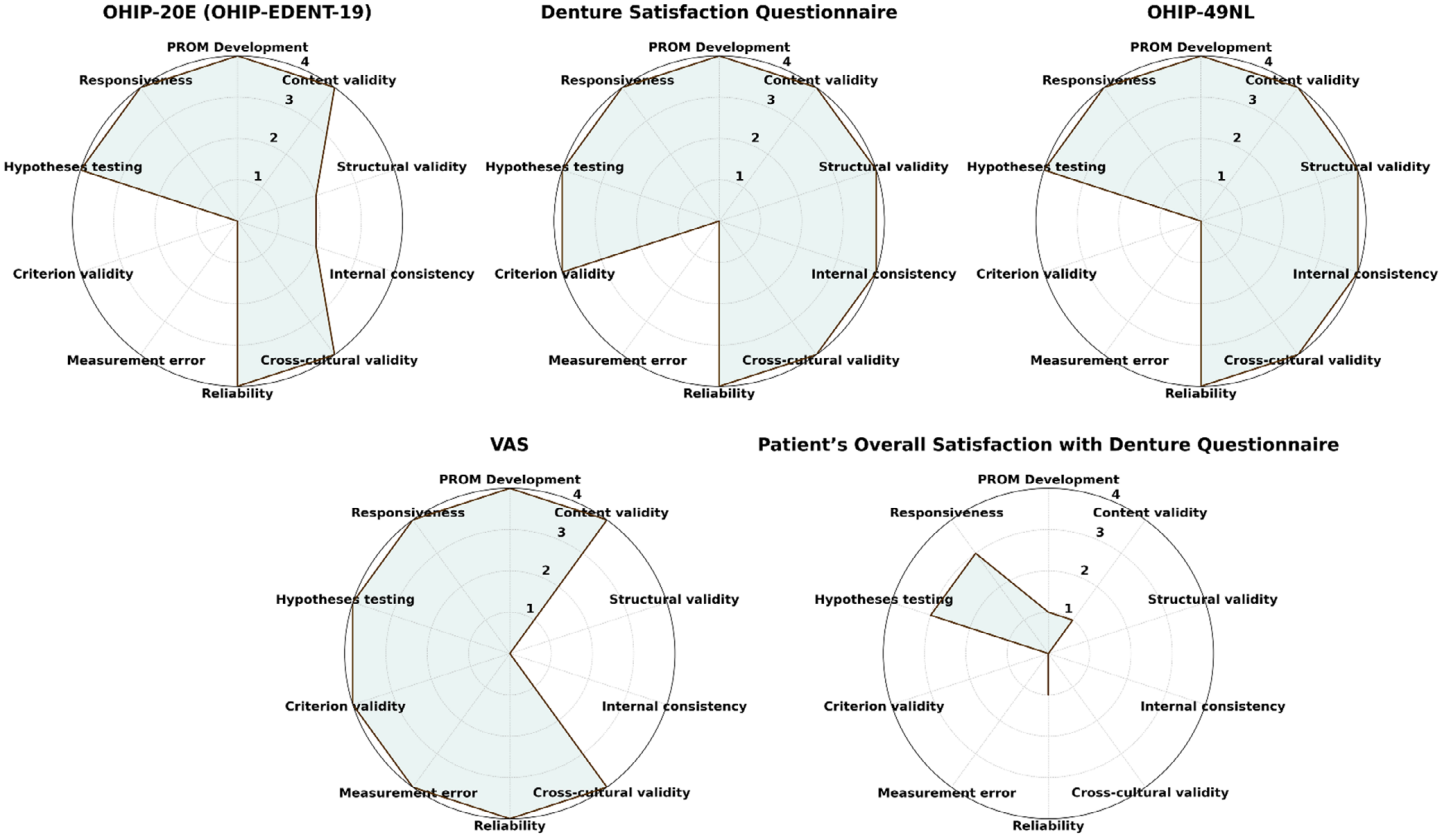
Radar plot of the assessment of included PROMs via COSMIN analysis. In order to visualize the radar plots for each PROM, the COSMIN score of each domain was converted to a numeric value from 0 = inadequate to 4 = very good. The larger green radar area corresponds to a higher validity and quality of the PROM (OHIP, Oral Health Impact Profile; VAS, Visual Analogue Scale).

This systematic review included the evaluation of six PROMs using the COSMIN checklist (as depicted in Figure 1). The PROMs analyzed were OHIP‐19E (OHIP‐EDENT‐19), OHIP‐49NL, VAS, Denture Satisfaction Questionnaire, and Patient Satisfaction and Change in Psychology Questionnaire. Table 4 presents the COSMIN checklist scores evaluating the methodological quality of each study and reporting the assessment of the measurement properties per PROM. Also, Figure 4 presents the radar plot of the COSMIN score of each of the identified PROMs.

- *Structural validity* was evaluated in three of the PROMs, with OHIP‐49NL and the Denture Satisfaction Questionnaire scoring “very good,” indicating robust factor structures. The OHIP‐19E had a “doubtful” rating, reflecting some concerns in its factor structure, while structural validity was not applicable to VAS, Patient's Overall Satisfaction with Denture Questionnaire, and the Patient Satisfaction and Change in Psychology Questionnaire due to their single‐item nature or ad‐hoc design.
- *Reliability* was assessed for all PROMs except for the Patient Satisfaction and Change in Psychology Questionnaire and the Patient's Overall Satisfaction with Denture, which were rated as “inadequate.” The OHIP‐49NL, VAS, and Denture Satisfaction Questionnaire all received “very good” ratings, indicating high stability and consistency over time. The reliability of these PROMs was generally evaluated using the Intraclass Correlation Coefficient (ICC).
- *Content validity* was consistently evaluated across all studies, with OHIP‐19E, OHIP‐49NL, VAS, and the Denture Satisfaction Questionnaire receiving “very good” ratings. These high ratings indicate that the items within these PROMs adequately cover the relevant content domains. Patients' Overall Satisfaction with Denture and the Patient Satisfaction and Change in Psychology Questionnaire were rated as “inadequate,” highlighting the need for more comprehensive development and validation processes.
- *Internal consistency* was relevant for the OHIP‐19E, OHIP‐49NL, and the Denture Satisfaction Questionnaire, with OHIP‐49NL and the Denture Satisfaction Questionnaire scoring “very good,” demonstrating high inter‐item correlation measured by Cronbach's α. The OHIP‐19E received a “doubtful” rating, indicating potential issues with the homogeneity of its items.
- *Cross‐cultural validity* was assessed in studies involving OHIP‐19E and OHIP‐49NL, both of which scored “very good,” showing that these PROMs perform consistently across different cultural contexts. The remaining PROMs did not provide sufficient data to evaluate cross‐cultural validity, marking them as “not applicable.”
- *Hypotheses testing for construct validity* was performed for all PROMs, except the Patient Satisfaction and Change in Psychology Questionnaire and the Patient's Overall Satisfaction with Denture, which received “inadequate” ratings. The other PROMs (OHIP‐19E, OHIP‐49NL, VAS, and Denture Satisfaction Questionnaire) all scored “very good,” demonstrating strong evidence supporting their construct validity.
- *Responsiveness*, which measures the ability of a PROM to detect change over time, was rated “very good” for OHIP‐19E, OHIP‐49NL, VAS, and the Denture Satisfaction Questionnaire, indicating their effectiveness in detecting clinically meaningful changes. The Patient Satisfaction and Change in Psychology Questionnaire and the Patient's Overall Satisfaction with Denture were rated as “adequate.”
- *Measurement error* was not applicable in most cases due to the lack of specific error metrics like Standard Error of Measurement (SEM) and Minimal Detectable Change (MDC) in the studies.

**TABLE 4:** The COSMIN analysis results for each of the included PROMs and their corresponding score.

PROM/references used	Domain									
	PROM development	Content validity	Structural validity	Internal consistency	Cross‐cultural validity/measurement invariance	Reliability	Measurement error	Criterion validity	Hypotheses testing for construct validity	Responsiveness
OHIP‐19 (OHIP‐EDENT‐19) (Allen and Locker 2002)	Very good	Very good	Doubtful	Doubtful	Very good	Very good	Not applicable	Not applicable	Very good	Very good
OHIP‐49NL (van der Meulen et al. 2008)	Very good	Very good	Very good	Very good	Very good	Very good	Not applicable	Not applicable	Very good	Very good
VAS (Huskisson 1974; McCormack et al. 1988)	Very good	Very good	Not applicable	Not applicable	Very good	Very good	Very good	Very good	Very good	Very good
Denture Satisfaction Questionnaire (Vervoorn et al. 1988; Osman et al. 2018; Santucci et al. 2014)	Very good	Very good	Very good	Very good	Very good	Very good	Not applicable	Very good	Very good	Very good
Patient's overall satisfaction with denture (Slot et al. 2016, 2023)	Inadequate	Inadequate	Not applicable	Not applicable	Not applicable	Inadequate	Not applicable	Not applicable	Inadequate	Adequate
Patient’ Satisfaction and Change of Psychology” questionnaire	Inadequate	Inadequate	Not applicable	Not applicable	Not applicable	Inadequate	Not applicable	Not applicable	Inadequate	Adequate

*Note:* The colors indicate the methodological quality rating for each measurement property: Green = Very good, Light red = Doubtful, Red = Inadequate, and Yellow = Not applicable.

Abbreviations: OHIP, Oral Health Index Profile; PROM, patient‐reported outcome measure; VAS, Visual Analogue Scale.

## 4 Discussion

### 4.1 Overall Findings

In our review, 6 PROMs and 15 other outcome measures were detected from the 14 included articles. Moreover, only half of the included studies reported PROMs in addition to other outcome measures (Slot et al. 2016, 2023; Putters et al. 2019; Wu et al. 2015; Marković et al. 2022; Wortmann et al. 2019).

The diversity of PROMs used to assess PROs such as satisfaction with treatment and OHRQoL makes it challenging to compile evidence across studies, particularly in the context of systematic reviews and meta‐analyses. Standardizing these measures would facilitate more reliable comparisons and pooling of results, thereby supporting the identification of treatment modalities that patients find superior (Snyder et al. 2022). Only through this approach can we guide the clinical decision‐making to better meet patient needs. To address this, several guidelines and initiatives have been developed to promote the standardization of PROMs (Crossnohere et al. 2024). For instance, the US Food and Drug Administration (FDA) and the COSMIN initiative provide comprehensive frameworks for the development and validation of PROMs, advocating for their consistent use in clinical research (Jung et al. 2024). In addition, the Implant Dentistry Core Outcome Set and Measurement (ID‐COSM) international consensus report has taken initial steps to establish standardized core outcome sets for implant dentistry, including hard and soft tissue augmentation. It emphasizes the measurement of PROs such as comfort and overall satisfaction as mandatory in all implant dentistry clinical trials, but does not yet mandate these measures in clinical trials solely evaluating bone augmentation without implant placement, acknowledging them as important, though not essential (Tonetti et al. 2023).

When compared to other disciplines of medical and dental research, a wide range of prevalence of PROs reporting has been reported. Reuter‐Selbach et al. (2023) assessed the frequency of PROs reporting in the context of RCTs on root coverage procedures. They reported that 61.5% of the selected RCTs reported both PROs and other outcome measures. Similarly, Avila‐Ortiz et al. (2023) reviewed the PROs and some outcome measures used in the clinical studies on soft‐tissue augmentation around implants, where around 19% of PROs reporting was found in the included trials. Also, including both prospective and retrospective studies, Tonetti et al. found that the overall rate of PROs reporting in bone preservation or augmentation procedures in the oral cavity was 15.7% (Tonetti et al. 2023). The results of our study showed a frequency of 50% with respect to PROs reporting among the included studies. The higher rate in the study by Reuter‐Selbach et al. (2023), may be due to their more strict inclusion criteria in terms of the study designs (only RCTs), which results in consideration of more systematically performed clinical studies with a more comprehensive study protocol that mostly includes PROs within the main outcomes. Our study included both randomized and non‐randomized prospective clinical studies, which might contribute to a lower total frequency of PROs reporting. Nonetheless, when we only consider the RCTs in our study, the PROs reporting increases to 66.6%, making it closer to the study by Reuter‐Selbach et al. In contrast, the systematic review by Avila‐Ortiz et al., which was undertaken in the context of the ID‐COSM international consensus report, was based on a similar inclusion criterion to that of our study (Avila‐Ortiz et al. 2023).

Nevertheless, a significant difference can be noted (50% vs. 19%) in the frequency of PROs reporting within the included studies. One can attribute this to the fact that the context of full‐arch implant rehabilitation (in our study) may necessitate PROs assessment more than soft tissue grafting around implants, as it plays a considerably more important role in patients' quality of life. Moreover, within a more similar focus on the rehabilitation of full‐arch edentulism with fixed or removable dentures retained by dental implants, Messias et al., in a paper commissioned for the ID‐COSM international consensus report, reported that approximately 34.4% of the included studies provided PROs reporting (Messias et al. 2023). However, it should be noted that they defined a broader eligibility criterion (retrospective cohorts, case series, etc.). Overall, it can be postulated that there has been a variable range in the consideration of PROs in dental research.

Considering that the main intervention in the included studies was ARA, there is a notable gap regarding the limited number of clinical measures directly associated with ARA outcomes. The most commonly reported ClinROs in bone preservation or augmentation in the oral cavity, according to Shi et al.'s review of 783 retrospective and prospective clinical studies, is the rate of complications (59.3%), implant survival (58.2%), 3D radiographic bone gain/change (30%), marginal bone level (MBL; 29%), and histological outcomes (25.5%), with implant survival also reported separately in 13.0% (Shi et al. 2023). These findings are consistent with the results of this study, with the most frequently reported ClinRO being implant survival (50%), peri‐implant bone changes (29%), CBCT linear and/or volumetric changes (33%), and histological assessment (25.8%).

Some of the studies included final prostheses‐related PROMs which assess patients' perceptions about function and esthetic or chewing ability. However, it should be borne in mind that, when ridge augmentation is the main intervention of a clinical study, prior to assessing prosthesis‐related PROs, assessing patients' experience during the early post‐operative phase is crucial. Significant pain/discomfort may be associated with large grafting procedures or harvesting of autogenous bone (Yu et al. 2023).

Another important argument is that some of the implemented ClinRO measures are directly affected by the type of grafting procedure. For instance, the study by Putters et al. (2018), which used calvarial bone or iliac crest as autogenous grafts, reported “hospitalization period” as one of the main ClinRO measures. This is clearly related only to augmentation procedures involving an extraoral donor site and, therefore, does not apply to other studies.

The COSMIN analysis highlights the importance of using well‐validated tools to ensure reliable and accurate measurement of PROs in dental research and practice (Prinsen et al. 2018; Mokkink et al. 2018; Terwee et al. 2018). The systematic review revealed that while several PROMs like *OHIP‐49NL*, *VAS*, and the *Denture Satisfaction Questionnaire* exhibit strong psychometric properties, making them reliable and valid tools for assessing PROs in dental research, the *OHIP‐19E* showed some limitations in structural validity and internal consistency but was otherwise robust. Unfortunately, to the best of the authors' knowledge, other PROMs such as the *Patient's Overall Satisfaction with Denture* and the *Patient Satisfaction and Change in Psychology Questionnaire* require significant improvement and further validation. In the systematic review by Messias et al. (2023), on PROMs in full‐arch rehabilitations, the edentulous version of the OHIP questionnaire (*OHIP‐19E/OHIP‐Edent*), the *chewing ability questionnaire*, and the *Denture Satisfaction Questionnaire* were used in 38.2%, 9%, and 11.7% of the included total studies. All these three measures in our study were used in 50% of the included trials. This difference is mainly due to the significant difference in the number of included trials (421 versus 15 in our study). However, it is important to note that a full overlap of the identified PROMs between our study and Messias et al.'s study (which is the only available systematic review that is directly related to PROMs in edentulous maxilla rehabilitation), confirms that these measures are consistently relevant and widely accepted in assessing patient outcomes in this specific context. This alignment reinforces the validity of our findings and highlights the importance of these PROMs in evaluating the effectiveness of full‐arch rehabilitations.

### 4.2 Limitations

Despite the comprehensive nature of this systematic review, several limitations should be acknowledged. Firstly, the relatively small number of included studies (14), presenting 11 unique trials, limits the generalizability of our findings, which may be due to the limited number of existing studies in the literature on this specific indication (maxillary arch augmentation in edentulous maxillae). The narrow timeframe (2013–2024) further restricts the scope of the review, potentially overlooking earlier relevant studies. We specifically chose those past 10 years follow‐up and only prospective studies to capture the most updated and reliable PROMs.

### 4.3 Recommendations for Future Research

Based on the findings of this systematic review and the COSMIN analysis, the following recommendations for future research are proposed:

- *Improvement of PROMs*: The *Patient Satisfaction and Change in Psychology Questionnaire* and the *Patient's Overall Satisfaction with Denture* showed significant limitations in validity and reliability. Future research should focus on improving the structural validity and internal consistency of these PROMs to ensure they accurately reflect patient experiences and outcomes.
- *Incorporation of ARA‐specific ClinRO measures*: Studies focusing on ARA should incorporate ClinRO measures that directly relate to ARA outcomes. Specific measures such as clinical bone gain, bone quality, histological outcomes, and patient morbidity related to the donor site need to be systematically included and reported to provide a comprehensive assessment of these interventions.
- *Early healing and long‐term PROMs*: In addition to final prosthetic‐related PROMs, there is a need for early healing PROMs. Ideally, there should be an initial set of PROMs assessing pain, discomfort, and overall experience immediately after ARA in the edentulous ridge. Subsequently, in the long term, PROMs should assess chewing function, esthetics, overall psychological satisfaction, and the impact of the final prosthesis on the patient's quality of life. This dual‐phase approach will provide a more holistic understanding of patient outcomes throughout the treatment process.
- *Standardization of PROMs and ClinRO reporting*: A standardized protocol for regularly using tools in studies involving ARA in the fully edentulous maxilla is clearly needed. The lack of standardization hampers comparability and the synthesis of evidence. For policymakers and researchers, it is crucial to establish and implement standardized tools for both ClinROs and PROs to ensure consistency and reliability across studies.

## 5 Conclusions

PROs were reported in only 50% of the included studies. In patients undergoing alveolar ridge augmentation of the fully edentulous maxilla, the OHIP‐49NL and Denture Satisfaction Questionnaire showed strong validity, while the Patient Satisfaction and Change in Psychology Questionnaire and Patient's Overall Satisfaction with Denture demonstrated substantial limitations. Standardized protocols for PROMs and ClinRO measures are essential to improve study consistency and reliability.

## Author Contributions

**Muhammad H. A. Saleh:** conceptualization, project administration, writing – review and editing. **Hamoun Sabri:** methodology, formal analysis, writing – original draft, visualization. **Parham Hazrati:** formal analysis, data curation. **Hom‐Lay Wang:** supervision, writing – review and editing.

## Conflicts of Interest

The authors declare no conflicts of interest.

## Supporting information


**Data S1:** Supporting Information.

## Data Availability

The original data regarding this study will be provided upon reasonable request from the corresponding author.
