# Supplementary material for: Outcome Measures in Alveolar Ridge Augmentation in the Edentulous Maxilla: A Systematic Review and COSMIN Analysis
Source: Clin Oral Implants Res. 2026 Feb 24;37(Suppl 30):S372–94. doi: 10.1111/clr.70060 (PMC12930129; doi:10.1111/clr.70060)
Supplement: Supplementary file 1 — Data S1: Supporting Information. [file CLR-37-S372-s001.docx]

**Supplementary Materials**

**Study title:** Patient-reported outcome measures and clinician-reported outcomes in alveolar ridge augmentation in the edentulous maxilla: A systematic review and COSMIN analysis.

Contents

[**Appendix Table 1.** The PRISMA 2020 statement checklist for systematic reviews. 2](#_Toc199877487)

[**Appendix Figure 1.** The flowchart for COSMIN analysis for PROMs according to the latest guidelines of this methodology. 5](#_Toc199877488)

[**Appendix Table 2.** The excluded studies, following full-text evaluation, and their reasons for exclusion. 6](#_Toc199877489)

# **Appendix Table 1.** The PRISMA 2020 statement checklist for systematic reviews.

| **Section and Topic** | **Item #** | **Checklist item** | **Location where item is reported** |
| --- | --- | --- | --- |
| **TITLE** | | |  |
| Title | 1 | Identify the report as a systematic review. | 1 |
| **ABSTRACT** | | |  |
| Abstract | 2 | See the PRISMA 2020 for Abstracts checklist. | 1,2 |
| **INTRODUCTION** | | |  |
| Rationale | 3 | Describe the rationale for the review in the context of existing knowledge. | 3.4 |
| Objectives | 4 | Provide an explicit statement of the objective(s) or question(s) the review addresses. | 4 |
| **METHODS** | | |  |
| Eligibility criteria | 5 | Specify the inclusion and exclusion criteria for the review and how studies were grouped for the syntheses. | 5 |
| Information sources | 6 | Specify all databases, registers, websites, organisations, reference lists and other sources searched or consulted to identify studies. Specify the date when each source was last searched or consulted. | 5,6 |
| Search strategy | 7 | Present the full search strategies for all databases, registers and websites, including any filters and limits used. | 6 |
| Selection process | 8 | Specify the methods used to decide whether a study met the inclusion criteria of the review, including how many reviewers screened each record and each report retrieved, whether they worked independently, and if applicable, details of automation tools used in the process. | 6,7 |
| Data collection process | 9 | Specify the methods used to collect data from reports, including how many reviewers collected data from each report, whether they worked independently, any processes for obtaining or confirming data from study investigators, and if applicable, details of automation tools used in the process. | 6,7 |
| Data items | 10a | List and define all outcomes for which data were sought. Specify whether all results that were compatible with each outcome domain in each study were sought (e.g. for all measures, time points, analyses), and if not, the methods used to decide which results to collect. | p. 7,8, table 1 |
|  | 10b | List and define all other variables for which data were sought (e.g. participant and intervention characteristics, funding sources). Describe any assumptions made about any missing or unclear information. | Table 1 |
| Study risk of bias assessment | 11 | Specify the methods used to assess risk of bias in the included studies, including details of the tool(s) used, how many reviewers assessed each study and whether they worked independently, and if applicable, details of automation tools used in the process. | p. 6 and 9 |
| Effect measures | 12 | Specify for each outcome the effect measure(s) (e.g. risk ratio, mean difference) used in the synthesis or presentation of results. | p. 7-10 |
| Synthesis methods | 13a | Describe the processes used to decide which studies were eligible for each synthesis (e.g. tabulating the study intervention characteristics and comparing against the planned groups for each synthesis (item #5)). | P 6-10 |
|  | 13b | Describe any methods required to prepare the data for presentation or synthesis, such as handling of missing summary statistics, or data conversions. | P 6-10 |
|  | 13c | Describe any methods used to tabulate or visually display results of individual studies and syntheses. | P 6-10 |
|  | 13d | Describe any methods used to synthesize results and provide a rationale for the choice(s). If meta-analysis was performed, describe the model(s), method(s) to identify the presence and extent of statistical heterogeneity, and software package(s) used. | p. 7-9 |
|  | 13e | Describe any methods used to explore possible causes of heterogeneity among study results (e.g. subgroup analysis, meta-regression). | p. 7-9 |
|  | 13f | Describe any sensitivity analyses conducted to assess robustness of the synthesized results. | - |
| Reporting bias assessment | 14 | Describe any methods used to assess risk of bias due to missing results in a synthesis (arising from reporting biases). | P 7-9 |
| Certainty assessment | 15 | Describe any methods used to assess certainty (or confidence) in the body of evidence for an outcome. | p. 10 |
| **RESULTS** | | |  |
| Study selection | 16a | Describe the results of the search and selection process, from the number of records identified in the search to the number of studies included in the review, ideally using a flow diagram. | p. 9,10 Fig. 1 |
|  | 16b | Cite studies that might appear to meet the inclusion criteria, but which were excluded, and explain why they were excluded. | Appendix C |
| Study characteristics | 17 | Cite each included study and present its characteristics. | p.9-11 Table 1 |
| Risk of bias in studies | 18 | Present assessments of risk of bias for each included study. | p.14,  Figures 5-8 |
| Results of individual studies | 19 | For all outcomes, present, for each study: (a) summary statistics for each group (where appropriate) and (b) an effect estimate and its precision (e.g. confidence/credible interval), ideally using structured tables or plots. | p. 12 and 16  Figure 2-4  Tables 3-5 |
| Results of syntheses | 20a | For each synthesis, briefly summarise the characteristics and risk of bias among contributing studies. | p. 14 |
|  | 20b | Present results of all statistical syntheses conducted. If meta-analysis was done, present for each the summary estimate and its precision (e.g. confidence/credible interval) and measures of statistical heterogeneity. If comparing groups, describe the direction of the effect. | p. 11-14  Figures 2-4 |
|  | 20c | Present results of all investigations of possible causes of heterogeneity among study results. | Appendix D-F |
|  | 20d | Present results of all sensitivity analyses conducted to assess the robustness of the synthesized results. | - |
| Reporting biases | 21 | Present assessments of risk of bias due to missing results (arising from reporting biases) for each synthesis assessed. | - |
| Certainty of evidence | 22 | Present assessments of certainty (or confidence) in the body of evidence for each outcome assessed. | - |
| **DISCUSSION** | | |  |
| Discussion | 23a | Provide a general interpretation of the results in the context of other evidence. | p. 15 |
|  | 23b | Discuss any limitations of the evidence included in the review. | p. 17 |
|  | 23c | Discuss any limitations of the review processes used. | p. 17 |
|  | 23d | Discuss implications of the results for practice, policy, and future research. | p. 18, 19 |
| **OTHER INFORMATION** | | |  |
| Registration and protocol | 24a | Provide registration information for the review, including register name and registration number, or state that the review was not registered. | p. 4 |
|  | 24b | Indicate where the review protocol can be accessed, or state that a protocol was not prepared. | p. 4 |
|  | 24c | Describe and explain any amendments to information provided at registration or in the protocol. | - |
| Support | 25 | Describe sources of financial or non-financial support for the review, and the role of the funders or sponsors in the review. | p. 19 |
| Competing interests | 26 | Declare any competing interests of review authors. | p. 19 |
| Availability of data, code and other materials | 27 | Report which of the following are publicly available and where they can be found: template data collection forms; data extracted from included studies; data used for all analyses; analytic code; any other materials used in the review. | - |

# **Appendix Figure 1.** The flowchart for COSMIN analysis for PROMs according to the latest guidelines of this methodology.^1^


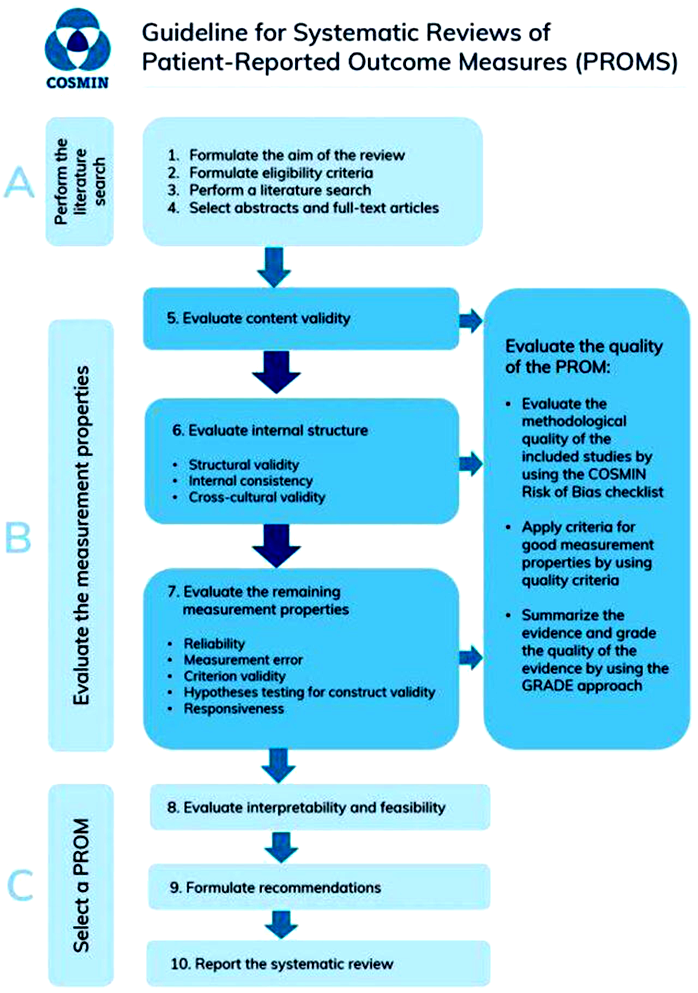


# **Appendix Table 2.** The excluded studies, following full-text evaluation, and their reasons for exclusion.

| **Author, Year** | **Reason for exclusion** |
| --- | --- |
| Weingart, 2000**^2^** | Review. |
| Att, 2009**^3^** | Review. |
| Lambert, 2009**^4^** | Review. |
| Esposito, 2013**^5^** | Review. |
| Taschieri, 2014**^6^** | Retracted article. |
| Pereira, 2015**^7^** | Pooling fully and partially edentulous patients. |
| Mertens, 2017**^8^** | Retrospective study. |
| Verhamme, 2017**^9^** | Digital accuracy study. |
| Verhamme, 2017**^10^** | Digital accuracy study. |
| Schnutenhaus, 2018**^11^** | Technical note. |
| Elhadidi, 2019**^12^** | Less than 10 patients. |
| Mangano, 2019**^13^** | No bone augmentation performed. |
| Toia, 2020**^14^** | No bone augmentation performed. |
| Yousif, 2020**^15^** | Less than 10 patients. |
| Hamilton, 2021**^16^** | No PROMs and CROMs reported; CBCT analysis. |
| Rossi, 2021**^17^** | Less than 10 patients. |
| Nilius, 2022**^18^** | Case report. |
| Reyes, 2022**^19^** | Less than 10 patients. |
| Abaza, 2023**^20^** | Partial edentulism. |
| Lotfazar, 2024**^21^** | Partial edentulism. |
| Masoomi, 2024**^22^** | Finite element analysis with no human subjects. |
| Nagib, 2024**^23^** | No bone augmentation performed. |
| Wang, 2024**^24^** | Less than 10 patients. |

**References**

1. Prinsen CAC, Mokkink LB, Bouter LM, et al. COSMIN guideline for systematic reviews of patient-reported outcome measures. *Qual Life Res* 2018;27:1147–1157.

2. Weingart D, ten Bruggenkate CM. Treatment of fully edentulous patients with ITI implants. *Clin Oral Implants Res* 2000;11 Suppl 1:69–82.

3. Att W, Bernhart J, Strub JR. Fixed rehabilitation of the edentulous maxilla: possibilities and clinical outcome. *J Oral Maxillofac Surg* 2009;67:60–73.

4. Lambert FE, Weber HP, Susarla SM, Belser UC, Gallucci GO. Descriptive analysis of implant and prosthodontic survival rates with fixed implant-supported rehabilitations in the edentulous maxilla. *J Periodontol* 2009;80:1220–1230.

5. Esposito M, Worthington HV. Interventions for replacing missing teeth: dental implants in zygomatic bone for the rehabilitation of the severely deficient edentulous maxilla. *Cochrane Database Syst Rev* 2013;2013:Cd004151.

6. Taschieri S, Corbella S, Francetti L, Del Fabbro M. Horizontal bone augmentation in full-arch maxillary implant-supported restorations: a preliminary clinical report. *Implant Dent* 2014;23:753–759.

7. Pereira E, Messias A, Dias R, Judas F, Salvoni A, Guerra F. Horizontal Resorption of Fresh-Frozen Corticocancellous Bone Blocks in the Reconstruction of the Atrophic Maxilla at 5 Months. *Clin Implant Dent Relat Res* 2015;17 Suppl 2:e444–458.

8. Mertens C, Freier K, Engel M, Krisam J, Hoffmann J, Freudlsperger C. Reconstruction of the severely atrophic edentulous maxillae with calvarial bone grafts. *Clin Oral Implants Res* 2017;28:749–756.

9. Verhamme LM, Meijer GJ, Soehardi A, Bergé SJ, Xi T, Maal TJJ. An accuracy study of computer-planned implant placement in the augmented maxilla using osteosynthesis screws. *Int J Oral Maxillofac Surg* 2017;46:511–517.

10. Verhamme LM, Meijer GJ, Bergé SJ, et al. An Accuracy Study of Computer-Planned Implant Placement in the Augmented Maxilla Using Mucosa-Supported Surgical Templates. *Clin Implant Dent Relat Res* 2015;17:1154–1163.

11. Schnutenhaus S, Neveling U, Luthardt RG. Digital implantological workflow for a CAD/CAM immediate long-term temporary restoration for the edentulous maxilla. *Int J Comput Dent* 2018;21:133–146.

12. Elhadidi M, Aldahouk A, Shawky M, Elbehairy MS, Atef M, El-Gengehi M. Computer-guided calvarial mono-cortical bone blocks harvest: A novel approach for three-dimensional alveolar reconstruction of atrophic maxilla. *Clin Implant Dent Relat Res* 2019;21:85–93.

13. Mangano F, Mangano C, Margiani B, Admakin O. Combining Intraoral and Face Scans for the Design and Fabrication of Computer-Assisted Design/Computer-Assisted Manufacturing (CAD/CAM) Polyether-Ether-Ketone (PEEK) Implant-Supported Bars for Maxillary Overdentures. *Scanning* 2019;2019:4274715.

14. Toia M, Stocchero M, Corrà E, Becktor JP, Wennerberg A, Cecchinato D. Fixed full-arch maxillary prostheses supported by four versus six implants with a titanium CAD/CAM milled framework: 3-year multicentre RCT. *Clin Oral Implants Res* 2021;32:44–59.

15. Yousif A, Raghoebar GM, Putters TF, Vissink A, Schortinghuis J. Calvarial bone grafts to augment the alveolar process in partially dentate patients: a prospective case series. *Int J Implant Dent* 2020;6:57.

16. Hamilton A, Jamjoom FZ, Alnasser M, Starr JR, Friedland B, Gallucci GO. Tilted versus axial implant distribution in the posterior edentulous maxilla: A CBCT analysis. *Clin Oral Implants Res* 2021;32:1357–1365.

17. Rossi F, Tuci L, Ferraioli L, et al. Two-Year Follow-Up of 4-mm-Long Implants Used as Distal Support of Full-Arch FDPs Compared to 10-mm Implants Installed after Sinus Floor Elevation. A Randomized Clinical Trial. *Int J Environ Res Public Health* 2021;18.

18. Nilius M, Mueller C, Nilius MH, Haim D, Weiland B, Lauer G. Advanced backward planning with custom-milled individual allogeneic block augmentation for maxillary full-arch osteoplasty and dental implantation:a 3-year follow-up. *Cell Tissue Bank* 2022;23:335–345.

19. Reyes Á JS, de Moura MB, Cartelli CA, Bernardes SR, Trojan LC, Thomé G. Prospective Evaluation of Bone Remodeling in Full-Arch Rehabilitations with Morse Taper Implants: A Case Series Study with One-Year Follow-Up. *J Long Term Eff Med Implants* 2022;32:83–92.

20. Abaza A, Abbas WM, Khalik DMA, El Din NHK. Horizontal Ridge Augmentation of the Atrophic Maxilla Using Pericardium Membrane Versus Titanium Mesh: A Clinical and Histologic Randomized Comparative Study. *Int J Oral Maxillofac Implants* 2023;38:451–461.

21. Lotfazar M, Amid R, Moscowchi A. Potentials of pure xenograft materials in maxillary ridge augmentation: A case series. *Saudi Dent J* 2024;36:187–191.

22. Masoomi F, Mahboub F. Stress distribution pattern in all-on-four maxillary restorations supported by porous tantalum and solid titanium implants using three-dimensional finite element analysis. *Eur J Transl Myol* 2024;34.

23. Nagib MA, Ibrahim AM, Abdel-Rahman FH, Hegazy SA, Habib A. Evaluation of Quality of Life and Satisfaction with Fixed Prostheses on Zygomatic Implants vs All-on-Four Concept: A Randomized Clinical Study. *J Contemp Dent Pract* 2024;25:141–147.

24. Wang S, Duan S, Chen R, Wang Z, Tang Y. Immediate loading in partially edentulous patients with fixed implant-supported restorations cases report. *Front Oral Health* 2024;5:1369494.
